# Osmolyte transporter expression is reduced in photoaged human skin: Implications for skin hydration in aging

**DOI:** 10.1111/acel.13058

**Published:** 2019-11-26

**Authors:** April R. Foster, Cecile El Chami, Catherine A. O'Neill, Rachel E. B. Watson

**Affiliations:** ^1^ Centre for Dermatology Research Faculty of Biology, Medicine and Health University of Manchester & Salford Royal NHS Foundation Trust Manchester Academic Health Science Centre Manchester UK; ^2^ NIHR Manchester Biomedical Research Centre Central Manchester University Hospitals NHS Foundation Trust Manchester UK

**Keywords:** aging, cell volume, human skin, osmolytes

## Abstract

Aging is characterized by the deterioration of tissue structure and function. In skin, environmental factors, for example, ultraviolet radiation (UVR), can accelerate the effects of aging such as decline in barrier function and subsequent loss of hydration. Water homeostasis is vital for all cellular functions and it is known that organic osmolyte transport is critical to this process. Therefore, we hypothesized that as we age, these tightly controlled physiological mechanisms become disrupted, possibly due to loss of transporter expression. We investigated this in vivo, using human skin samples from photoprotected and photoexposed sites of young and aged volunteers. We show a reduction in keratinocyte cell size with age and a downregulation of osmolyte transporters SMIT and TAUT with both chronic and acute UVR exposure. Single‐cell live imaging demonstrated that aged keratinocytes lack efficient cell volume recovery mechanisms possessed by young keratinocytes following physiological stress. However, addition of exogenous taurine significantly rescued cell volume; this was corroborated by a reduction in TAUT mRNA and protein in aged, as compared to young, keratinocytes. Collectively, these novel data demonstrate that human epidermal keratinocytes possess osmolyte‐mediated cell volume regulatory mechanisms, which may be compromised in aging. Therefore, this suggests that organic osmolytes—especially taurine—play a critical role in cutaneous age‐related xerosis and highlights a fundamental mechanism, vital to our understanding of the pathophysiology of skin aging.

## Graphical abstract

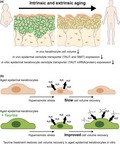

## 1 INTRODUCTION

Aging is a fundamental process of life in which there is a time‐dependent decline of physiological integrity and cellular function affecting all tissues (Gunn et al., 2015; López‐Otín, Blasco, Partridge, Serrano, & Kroemer, 2013; Makrantonaki et al., 2012). In skin, these changes can occur due to the passage of time (intrinsic aging) and may be accelerated by environmental factors, the principal one being exposure to ultraviolet radiation (extrinsic aging) (Jenkins, 2002; Pillai, Oresajo, & Hayward, 2005; Tsoureli‐Nikita, Watson, & Griffiths, 2006). As the major interface between the body and the environment, skin is exposed to many environmental factors and presents with the most visible signs of aging. Physical features such as wrinkling, laxity, hyperpigmentation, and dryness are used to clinically characterize aging (Flament et al., 2013; Jenkins, 2002; Kennedy et al., 2003). Another aspect of cutaneous aging is decline in barrier function, where increased epidermal fragility, thinning, and impaired barrier repair are thought to contribute to skin dryness or xerosis (Ghadially, Brown, Sequeira‐Martin, Feingold, & Elias, 1995; Kuehne et al., 2017).

The epidermis of skin is critical for controlling water homeostasis, which is fundamental for all cellular processes. Barrier function is provided by the *stratum corneum* and cell–cell junctions between epidermal keratinocytes which together control trans‐epidermal water loss (TEWL) (Brandner et al., 2002; Denda et al., 1998; Kirschner et al., 2013). However, the continuous exposure of skin to a dry surrounding environment can lead not only to loss of extracellular water, but also water loss from within the keratinocytes. Intracellular water leaves keratinocytes down a concentration gradient as the extracellular osmotic environment becomes hypertonic (Denda et al., 1998; Denda, Sokabe, Fukumi‐Tominaga, & Tominaga, 2007; El‐Chami, Haslam, Steward, & O'Neill, 2014; Verdier‐Sévrain & Bonté, 2007). The cellular control of water homeostasis is critical; cell shrinkage can lead to cell death and if this goes unresolved, it ultimately leads to tissue dehydration and, potentially organismal death. Therefore, important cellular mechanisms exist to maintain tight control of cell volume. One of the mechanisms used by cells involves the expression of naturally occurring compounds known as organic osmolytes, such as betaine, myoinositol, and taurine (El‐Chami et al., 2014; Strange, 2004). Such osmolytes are transported into cells under water stress and can accumulate at high concentration without adverse effect, thus preventing further water loss from cells. Conversely, if cells are under threat of excessive swelling, osmolytes are actively pumped out of cells. These mechanisms allow movement of osmolytes and water molecules across the cell membrane via transporters and initiate cell volume recovery in response to osmotic fluctuation (Burg & Ferraris, 2008; El‐Chami et al., 2014; Ito, Miyazaki, Schaffer, & Azuma, 2015; Kroemer et al., 2009).

Despite many advances in cutaneous cell physiology, there is a paucity of information regarding the molecular mechanisms which control water homeostasis and how skin aging impacts on this. The role of organic osmolytes and the respective transporters has been investigated in other major organs such as the kidney, where cells are exposed to a highly concentrated and changing osmotic environment (Wang & Bolen, 1997). Studies have shown that organic osmolytes act to counteract these changes, not only by stabilizing cell volume, but also via protein stabilization and exerting antioxidant effects (Burg & Ferraris, 2008; Burg, Ferraris, & Dmitrieva, 2007; Ito et al., 2015; Khan, Ahmad, Ahmad, & Kumar, 2010; Samuel et al., 2000). However, only a few studies have considered the role of organic osmolytes and their transporters in skin (Anderheggen et al., 2006; Grafe, Wohlrab, Neubert, & Brandsch, 2004; Janeke et al., 2003; Lobo, Alonso, Latorre, & Martín del Río, 2001; Warskulat, Brookmann, Reinen, & Häussinger, 2007; Warskulat, Reinen, Grether‐Beck, Krutmann, & Häussinger, 2004). Warskulat et al. (2004), and Warskulat et al. (2007) report that both normal human epidermal keratinocytes (NHEKs) and HaCaT cells are osmosensitive and express the betaine transporter, BGT‐1, sodium‐coupled myoinositol transporter (SMIT), and taurine transporter (TAUT) (Warskulat et al., 2007, 2004). However, in skin, only TAUT has been demonstrated in vivo and was shown to be expressed in the *stratum granulosum* and *stratum spinosum* of human epidermis (Janeke et al., 2003). Taurine, the substrate of TAUT, was also shown to be expressed in these epidermal layers in canine and rat skin (Anderheggen et al., 2006). Recent work by our group has also demonstrated the expression of BGT‐1 and TAUT in human skin in organ culture (El‐Chami, Haslam, Steward, Clausen, & O'Neill, 2015). However, the provenance of this skin was unknown making it difficult to make concrete conclusions on the localization of the transporters since this could be affected by exposure to the environment. In reconstructed epidermis, taurine improved epidermal barrier function by reducing TEWL (Anderheggen et al., 2006). These data support the hypothesis for a role for organic osmolytes and their transporters in water homeostasis of skin cells which may be compromised in aged skin. Improving the understanding of these molecular changes of human skin could encourage the development of novel approaches for prevention and treatment of skin conditions linked to hydration.

Therefore, this study aimed to characterize the expression and localization of osmolyte transporters, BGT‐1, HMIT, SMIT, and TAUT in intrinsically and extrinsically aged human skin and in skin exposed to a single dose of acute‐solar simulated radiation (SSR). We also aimed to understand osmoregulation in young and aged primary epidermal keratinocytes exposed to hyperosmotic stress and ultraviolet radiation (UVR) using single‐cell live imaging.

## 2 RESULTS

### 2.1 Organic osmolyte transporter proteins have distinct expression patterns in human skin

**Figure 1:**
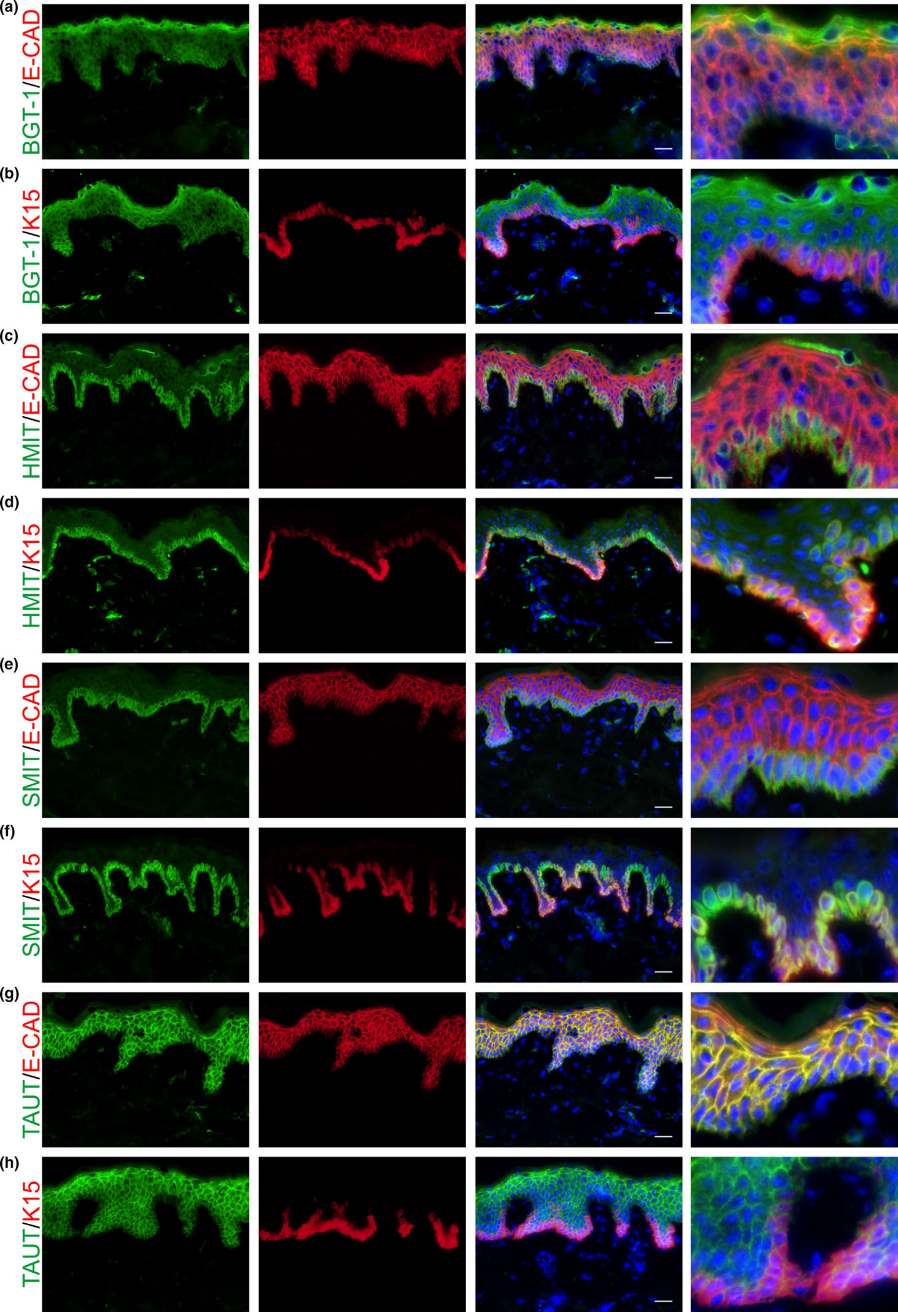
Organic osmolyte transporters are differentially expressed in the human epidermis in vivo. Organic osmolyte transporter protein expression was characterized in human photoprotected skin in vivo using dual immunofluorescence with the cell surface marker, e‐cadherin, and the basal cell marker, K15. Osmolyte transporters (a, b) BGT‐1, (c, d) HMIT, (e, f) SMIT, and (g, h) TAUT have distinct expression patterns in human skin. Scale bars = 20 µm. BGT‐1, betaine transporter; E‐CAD, e‐cadherin; HMIT, hydrogen‐coupled myoinositol transporter; K15, keratin 15; SMIT, sodium‐coupled myoinositol transporter; TAUT, taurine transporter

Firstly, characterization of key osmolyte transporters BGT‐1, HMIT, SMIT, and TAUT was performed using immunofluorescence in young, photoprotected buttock skin. This revealed distinct expression patterns of organic osmolyte transporter proteins in vivo in human skin (Figure 1). Betaine transporter, BGT‐1, was expressed as a gradient throughout the epidermis (Figure 1a‐b) and also in some dermal cells (Figure S1a‐b). Dual immunofluorescence of BGT‐1 with vimentin and cluster of differentiation 31 (CD31) confirmed this dermal staining with double‐positive immunoreactivity in dermal fibroblasts (Figure S1c‐f). Myoinositol is transported via either hydrogen‐coupled myoinositol transporter, HMIT, or sodium‐coupled myoinositol transporter, SMIT. Both HMIT and SMIT have the same protein expression pattern in human skin and are restricted to the *stratum basale* (Figure 1c‐f). Taurine transporter, TAUT, was expressed throughout the epidermis with lower expression in the *stratum basale* compared to the suprabasal layers (Figure 1g‐h). To assess the precise cellular localization of each of the four osmolyte transporters, dual immunofluorescence was carried out with the cell surface marker, e‐cadherin, and the basal cell marker, K15 (Figure 1). This revealed that all four transporters were membranous and that BGT‐1 was also cytoplasmic. Both HMIT and SMIT were co‐expressed by K15‐positive cells in the basal layer of the epidermis (Figure 1d and f). Finally, TAUT was co‐localized with e‐cadherin throughout the epidermis (Figure 1g).

### 2.2 Epidermal keratinocyte cell area and diameter are smaller in intrinsically and extrinsically aged human skin

**Figure 2:**
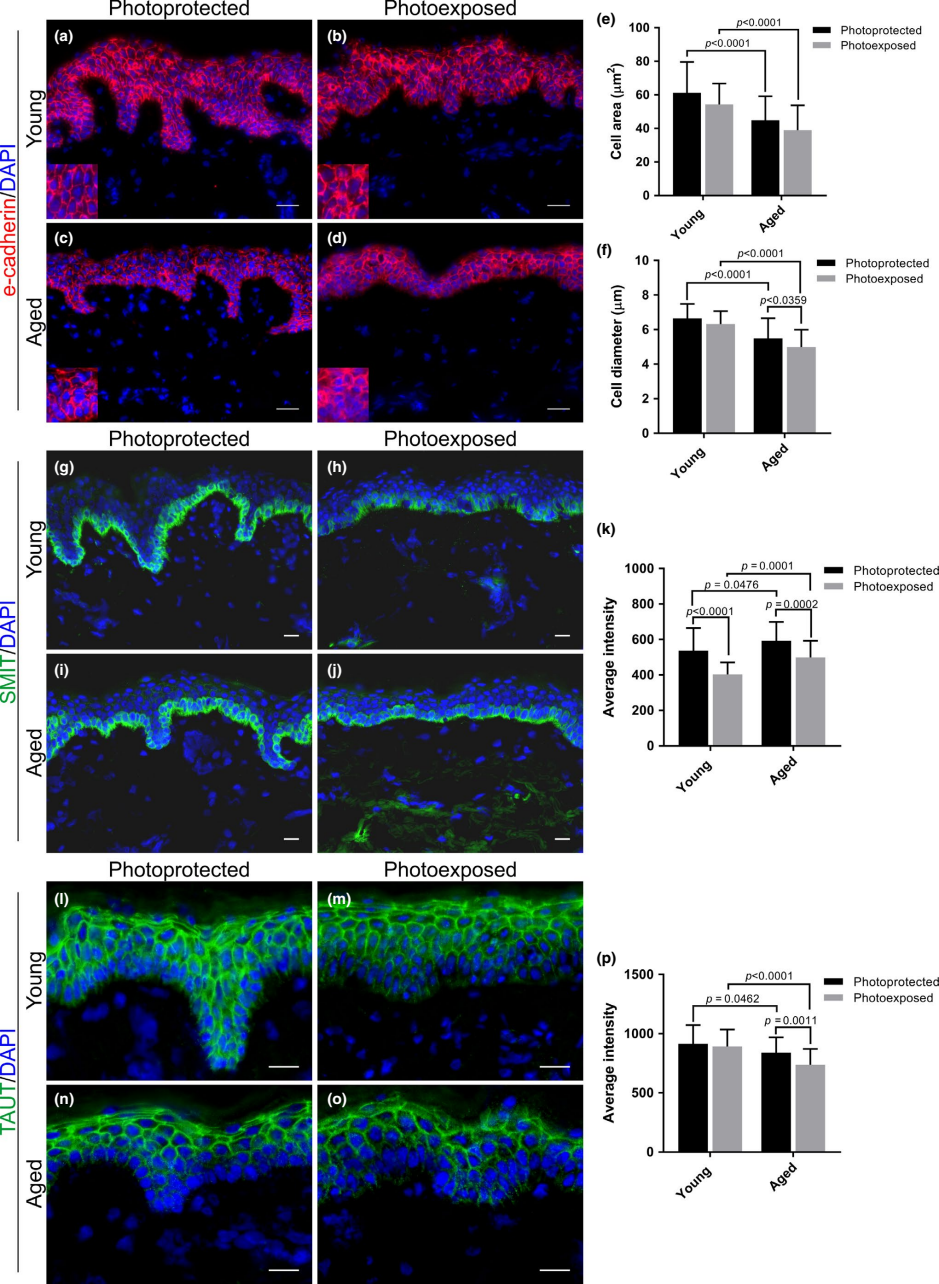
Epidermal keratinocytes cell size and osmolyte transporters SMIT and TAUT are decreased with either intrinsic or extrinsic aging. E‐cadherin immunofluorescence stain in (a, b) young and (c, d) aged photoprotected and photoexposed human skin. Morphological assessment of epidermal keratinocyte cell (e) area and (f) diameter in human skin. (g‐j) SMIT immunofluorescence in young and aged, photoprotected and photoexposed skin. (k) SMIT protein expression was significantly downregulated in photoexposed skin compared to photoprotected skin (young *p* < .0001 and aged *p* = .0002). (l‐o) TAUT immunofluorescence in young and aged, photoprotected and photoexposed skin. (j) TAUT protein expression was significantly lower in aged skin compared to young skin (photoprotected *p* = .0462 and photoexposed *p* < .0001). TAUT protein expression is also significantly downregulated in aged photoexposed skin compared to aged photoprotected skin (*p* = .0011). Data expressed as mean ± *SD* (two‐way ANOVA), young *n* = 5, aged *n* = 6. Scale bars = 20 µm. SMIT, sodium‐coupled myoinositol transporter; TAUT, taurine transporter

Skin samples were collected from young and aged volunteers from a photoprotected and a photoexposed anatomical site (buttock and forearm, respectively) (Table S1). To evaluate the effect of aging on morphological characteristics of epidermal keratinocytes, these samples were assessed for cell volume/area and cell diameter in the epidermis using immunofluorescence of e‐cadherin (Figure 2a‐f). This analysis revealed that cell area and diameter were altered with both intrinsic and extrinsic aging. Specifically, cell area was significantly smaller in aged photoprotected skin compared to young photoprotected skin (*p* < .0001) and also in aged photoexposed skin compared to young photoexposed skin (*p* < .0001) (Figure 2e). However, photoexposure per se did not affect cell area (Figure 2e). Similarly, cell diameter was significantly smaller in aged skin compared to young skin taken from both the photoprotected (*p* < .0001) and photoexposed (*p* < .0001) sites (Figure 2f). There was a small, but significant effect of photoexposure per se in aged photoexposed skin compared to aged photoprotected skin (*p* = .0359) (Figure 2f).

### 2.3 Protein expression of osmolyte transporters SMIT and TAUT is affected by age and photoexposure in human skin

Next, since keratinocyte cell size was affected by aging in human skin, we wanted to understand whether intrinsic and extrinsic aging also impacted upon protein expression of organic osmolyte transporters. To do this, immunofluorescence was performed for BGT‐1, HMIT, SMIT, and TAUT in young and aged, photoprotected, or photoexposed skin.

Analysis revealed that intrinsic aging had no significant effect on protein expression of BGT‐1. However, there was a significant difference in BGT‐1 expression between young and aged photoexposed skin (*p* = .0206) (Figure S2a‐e). HMIT protein expression was unchanged with age or anatomical location of skin (photoprotected vs. photoexposed) (Figure S2f‐j). By contrast, SMIT protein expression was significantly downregulated in photoexposed skin compared to skin which was photoprotected in both the young (*p* < .0001) and aged (*p* = .0002) cohorts (Figure 2g‐k). Interestingly, SMIT protein expression was higher in the epidermis of aged compared to young skin (photoprotected *p* = .0476 and photoexposed *p* = .0001) (Figure 2k). Analysis of TAUT revealed significantly lower protein expression in aged skin compared to young skin (*p* = .0462) (Figure 2l‐p). Furthermore, TAUT expression was lower in aged photoexposed skin compared to young photoexposed skin (*p* < .0001). TAUT protein expression was also significantly lower in aged photoexposed skin compared to aged photoprotected skin (*p* = .0011); however, photoexposure had no effect on TAUT in young skin (Figure 2n‐p). Furthermore, TAUT was expressed as a gradient in the epidermis of photoexposed skin.

### 2.4 HMIT, SMIT, and TAUT protein expression are downregulated 72 hr following acute‐solar simulated radiation (SSR)

**Figure 3:**
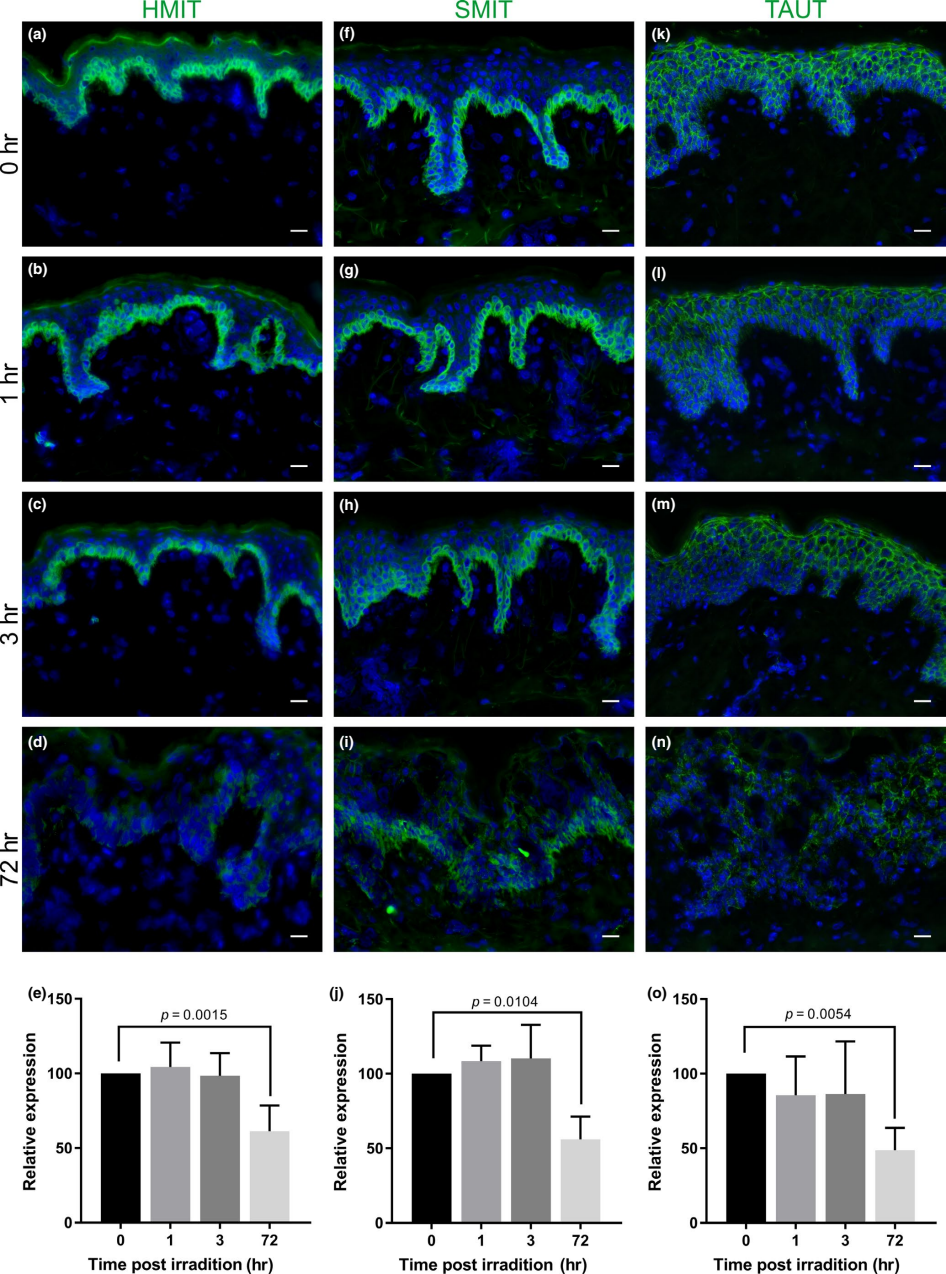
HMIT, SMIT, and TAUT are downregulated 72‐hr following exposure to acute‐solar simulated radiation (SSR) in vivo. Immunofluorescence for HMIT, SMIT, and TAUT was conducted in healthy photoprotected skin following acute irradiation (80 mJ) with solar simulated radiation (SSR) at (a, f, k) baseline (unirradiated control) and (b, g, l) 1‐hr, (c, h, m) 3 hr and (d, i, n) 72 hr following SSR exposure. Protein expression was significantly downregulated at 72‐hr post‐SSR for (e) HMIT (*p* = .0015), (j) SMIT (*p* = .0104), and (o) TAUT (*p* = .0054). Data expressed as mean ± *SD* (one‐way ANOVA), *n* = 5. Scale bars = 20µm. HMIT, hydrogen‐coupled myoinositol transporter; SMIT, sodium‐coupled myoinositol transporter; TAUT, taurine transporter

Analysis of young and aged skin samples revealed that chronic UVR exposure leads to a reduction in SMIT and TAUT protein expression (Figure 2) but no difference in HMIT protein expression (Figure S2). Next, we wanted to understand whether an acute UVR exposure could also affect these osmolyte transporter proteins in the skin in a similar manner to chronic UVR exposure. Healthy volunteers were exposed to 80 mJ (8x SED) full spectrum SSR on photoprotected buttock. Skin biopsies were taken prior to irradiation (baseline; 0 hr) then at 1, 3, and 72 hr post‐SSR, with immunofluorescence used to assess HMIT, SMIT, and TAUT protein expression following challenge (Figure 3).

HMIT protein expression was significantly downregulated 72 hr post‐SSR (*p* = .0015) (Figure 3a‐e), suggesting that there are differences in the effect of chronic versus acute SSR exposure on HMIT protein in human skin. Analysis revealed that SMIT is significantly downregulated in the epidermis 72‐hr following acute SSR exposure (*p* = .0104) (Figure 3f‐j). Similarly, analysis of TAUT expression identified a significant downregulation of protein in the epidermis 72 hr post‐SSR (*p* = .0054) (Figure 3k‐o).

### 2.5 Organic osmolytes improve cell volume regulatory mechanisms of aged NHEKs exposed to hyperosmolarity

The data presented thus far suggest that TAUT is affected by intrinsic aging and that organic osmolyte transporter proteins are susceptible to dysregulation by extrinsic factors. Therefore, in order to understand the possible functional implications of this, single‐cell live imaging was used to assess volume regulatory mechanisms of primary NHEKs from young and aged donors, in real time.

**Figure 4:**
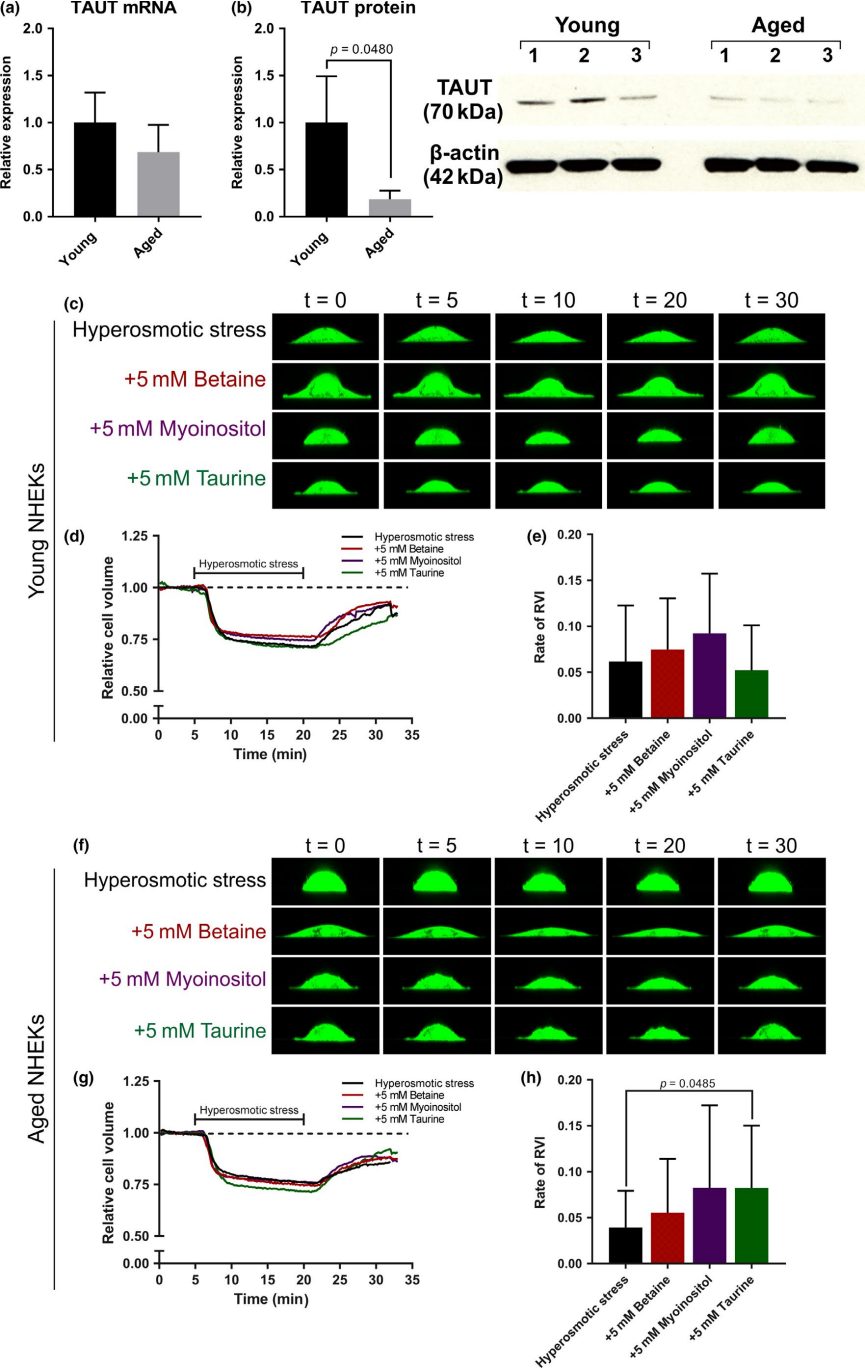
TAUT protein is reduced in aged NHEKs and taurine improves the rate of cell volume recovery in aged NHEKs exposed to hyperosmotic stress. (a) Relative mRNA expression of TAUT in young and aged NHEKs. (b) Western blot for TAUT protein expression revealed a significant downregulation in aged NHEKs compared to young NHEKs (*p* = .0480). Single‐cell live imaging was used to assess cell volume regulation in (c‐d) young and (f‐g) aged NHEKs through a 32‐min cycle of 5 min isosmotic conditions (300mOsm per L), 15 min hyperosmotic conditions (500mOsm per L), and 12 min isosmotic conditions in the absence and presence of the organic osmolytes. (e) The rate of RVI of young NHEKs. (h) For aged NHEKs, the rate of RVI significantly increased higher in the presence of taurine (*p* = .0485) compared to hyperosmotic stress alone. Data expressed as mean ± *SD* ((a,b) Student's *t* test, (e, h) one‐way ANOVA) (a, b) *n* = 3 young and 3 aged donors, (c‐e) *n* = 30 cells from three young and (f‐h) *n* = 30 cells from three aged donors. NHEKs, normal human epidermal keratinocytes; RVI, regulatory volume increase; t, time (in minutes); TAUT, taurine transporter

Initially, we confirmed the expression of organic osmolyte transporters in primary young and aged NHEKs using qRT–PCR and Western blotting. qRT–PCR revealed no significant difference in mRNA expression of any osmolyte transporters between young and aged cells (Figure 4a and S3a‐c). However, immunoblotting demonstrated a significant reduction in the protein expression of TAUT (but not BGT‐1, HMIT or SMIT) in aged NHEKs compared to young (*p = .048*) (Figure 4b and S3d‐f). Since these data reflect our observations in human skin, this suggests that primary keratinocytes isolated from young and aged individuals are a good model of osmolyte transporter expression in young and aged human skin.

Primary NHEKs were obtained from photoprotected anatomical sites to assess the functional impact of intrinsic aging on cell volume control. NHEKs were exposed to a cycle of 5 min isosmotic conditions (300 mOsm per L), 15 min hyperosmotic conditions (500 mOsm per L), and 12 min isosmotic conditions in the absence and presence of the organic osmolytes (Figure 4c and f).

Both young and aged NHEKs exposed to hyperosmotic stress underwent cell shrinkage within the first 5 min of application of stress (Figure 4d and g). Keratinocytes from young donors regained 88% cell volume when returned to isosmotic conditions after stress and this improved to 92% in the presence of betaine and myoinositol (Figure 4d and S4a), although this was not significant. The addition of betaine and myoinositol did not improve the rate of regulatory volume increase (RVI) of young NHEKs (Figure 4e). Keratinocytes from aged donors regained 84% cell volume when returned to isosmotic conditions after stress and 86%–89% in the presence of osmolytes (Figure 4g and S4b). Interestingly, the average rate of RVI in aged NHEKs was slower than young NHEKs. However, the rate of RVI in aged NHEKs was improved by the addition of betaine or myoinositol and significantly increased in the presence of taurine (*p* = .0485) (Figure 4h).

### 2.6 Young, but not aged NHEKs undergo cell volume changes following SSR exposure

Next, we wanted to understand whether keratinocyte volume regulatory mechanisms were affected by SSR exposure, since a downregulation in key osmolyte transporter proteins was observed in vivo in human skin with extrinsic aging and when exposed to acute UVR.

**Figure 5:**
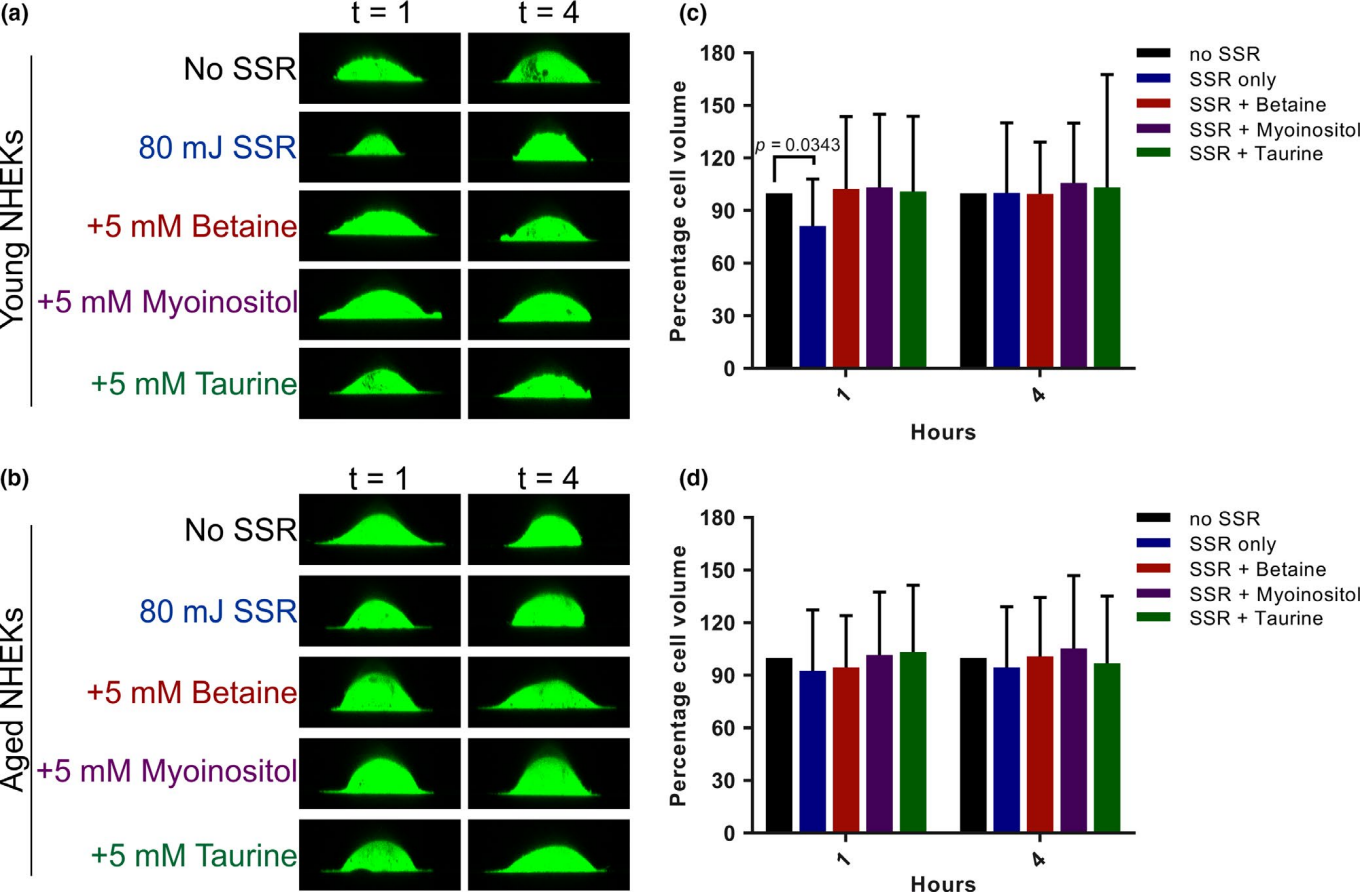
Aged NHEKs undergo minimal cell volume changes post‐SSR and have lower TAUT mRNA and protein expression than young NHEKs. Primary NHEKs from (a) young and (b) aged donors were exposed to 80 mJ SSR in the absence and presence of organic osmolytes. Cells were imaged at 1 and 4 hr following exposure, and percentage cell volume changes were calculated for (c) young and (d) aged NHEKs relative to the no SSR control. Data expressed as mean ± *SD* (one‐way ANOVA), *n* = 30 cells from three young and *n* = 30 cells from three aged donors. NHEKs, normal human epidermal keratinocytes; SSR, solar simulated radiation; t, time (in hours)

Young and aged NHEKs were exposed to 80 mJ SSR and then treated with betaine, myoinositol or taurine for 1 and 4 hr (Figure 5a and b). Single‐cell live imaging revealed that young NHEKs cell volume shrank by 19% at 1 hr post‐SSR compared to the no SSR control (*p* = .0343) (Figure 5c). However, the addition of betaine, myoinositol, and taurine did not prevent cell shrinkage at 1 hr post‐SSR (Figure 5c). Interestingly, cell volume of aged NHEKs did not change in response to SSR exposure at 1 hr or at 4 hr (Figure 5d), potentially because aged keratinocytes already have a reduced cell size and diameter (Figure 2a‐e). The application of betaine, myoinositol, or taurine had no effect on cell volume in SSR‐exposed aged NHEKs.

## 3 DISCUSSION

The precise regulation of cellular microenvironments is vital for fundamental cell processes such as cell cycling, DNA synthesis, and protein translation. The aging process and presence of physiological stressors can threaten these tightly controlled mechanisms, for example, altered cell volume regulation. Therefore, it is essential to understand how these mechanisms function in order to protect cells from stress, and how they are affected by aging. Previously, the role of organic osmolytes in human skin has been poorly understood. However, our study demonstrates that human epidermis possesses osmosensitive cell volume regulatory mechanisms which could have a role in skin hydration. Collectively, we have shown that in vivo, human skin expresses proteins required for osmolyte transport; that age and physiological stressors impact keratinocyte cell volume and transporter expression and; in vitro keratinocyte cultures, that addition of organic osmolytes can improve the rate of cell recovery following osmotic stress in aged cells.

Characterization of key organic osmolyte transporters in vivo in human skin demonstrated that BGT‐1, HMIT, SMIT, and TAUT have unique epidermal expression patterns, strongly suggesting that human keratinocytes possess an osmolyte strategy. The distinct expression patterns of these transporters were verified by dual immunofluorescence with cell surface marker, e‐cadherin, and basal cell marker, K15 (Figure 1). BGT‐1 is expressed in the dermis, as well as the epidermis (Figure 1a, 1b, and S1), which could indicate that human dermal fibroblasts can also respond to cell volume regulatory mechanisms. In agreement with this, it has been demonstrated that human dermal fibroblasts express BGT‐1, SMIT, and TAUT mRNA under normal conditions and that osmolyte uptake is affected following UVA exposure (Warskulat et al., 2008). HMIT and SMIT, both transporters of myoinositol, are expressed exclusively in the basal layer (Figure 1c‐f). It is known that myoinositol has a key role in osmoregulation and is a precursor for second messengers in different signaling pathways (Parthasarathy, Seelan, Tobias, Casanova, & Parthasarathy, 2006). Thus, the presence of these transporters in the basal layer raises the possibility that myoinositol could act as a second messenger between the epidermis and dermis potentially for osmoregulation. We also confirmed that TAUT is expressed as a gradient throughout the epidermis with highest expression at the uppermost living layers of the skin (Figure 1d), similar to the expression pattern that has been previously described (Janeke et al., 2003). However, we additionally demonstrate immunostaining for TAUT in the *stratum basale*.

Further investigations of human skin in vivo revealed that human epidermal keratinocytes were smaller with increasing age and photoexposure (Figure 2a‐f), while there is a loss of SMIT expression with photoexposure, both intrinsic and extrinsic aging lead to a significant downregulation of TAUT (Figure 2g‐p). SMIT and TAUT were also downregulated by a single acute dose of SSR, suggesting that they are susceptible to regulation by external insults (Figure 3f‐o). However, TAUT expression was not significantly different between photoprotected and photoexposed skin from young individuals, suggesting that the loss of TAUT expression in response to acute exposure to SSR can be recovered in young skin (Figure 2l‐p). An acute SSR insult also led to a downregulation of HMIT (Figure 3a‐e) which is not impacted by chronic UVR exposure in extrinsically aged skin (Figure S2), indicating that UVR‐induced changes to HMIT are not persistent. All these data suggest that the different transporters are impacted by intrinsic aging and extrinsic aging in specific ways. For some transporters such as TAUT, intrinsic aging may be a more important regulator of expression than aging in response to photoexposure. Taken together, these data demonstrate that aging and extrinsic factors impact SMIT and TAUT expression which could affect osmoregulation in human skin. A previous study by Warskulat et al., (2004), demonstrated increased mRNA expression of osmolyte transporters in response to a single dose of UVA or UVB in isolated keratinocytes. The apparent differences between their data and the data from our study are probably due to the different experimental models used—human skin in our study versus isolated keratinocytes. The difference in UV source could also be a factor. Our study analyzed transporter expression in human skin chronically exposed to sunlight over long periods of time, or in response to a single dose of acute SSR.

Single‐cell live imaging demonstrated cell volume regulatory mechanisms of young and aged NHEKs in real time. Analysis of the osmolyte transporter mRNA and protein in keratinocytes taken from young versus aged individuals revealed similar changes to those observed in human skin in vivo, that is, loss of TAUT protein expression in aged cells. This suggests that keratinocytes from aged individuals are a good model of transporter expression in aged human skin. When exposed to physiological stress (hyperosmolarity) aged NHEKs were slower to recover cell volume than young NHEKs (Figure 4 and Figure S4). Furthermore, addition of taurine improved the rate of RVI of aged NHEKs but not keratinocytes from young individuals. This could suggest that keratinocytes from young individuals have other mechanisms of osmotic control that they can employ, which may not be available in aged cells. Indeed, cellular water homeostasis is maintained by several transporters and their substrates, such as water itself and also inorganic osmolytes. Aquaporins (AQPs), for example, are responsible for water movement across cell membranes and have been shown to have a number of roles in skin, such as keratinocyte migration in wound healing, which is enabled by AQP3 (Hara‐Chikuma & Verkman, 2008; Ikarashi et al., 2017). Ikarashi et al., demonstrated that several AQPs were significantly lower in the skin of aged compared to young mice, as well as demonstrating age‐related changes to AQPs expression in other major organs. Therefore, this could suggest that other transporters involved in cell volume regulation should be investigated in cutaneous aging, along with the organic osmolyte strategy as we have demonstrated here.

We also show that SSR induced cell shrinkage in young but not aged NHEKs. Aged cells showed very minimal changes in cell volume in response to SSR, possibly because they are already smaller than young cells. Addition of osmolytes to young or aged cells did not significantly improve their volume regulatory response to SSR. This suggests that organic osmolytes may be used by keratinocytes depending on the stressor that the cells are exposed to. Taurine clearly improves cell volume recovery in response to increased osmolarity in cells from aged individuals, but not in response to acute SSR in cells from young individuals.

Taken together, these data suggest that age and environmental factors, specifically UVR, have a direct impact on osmolyte transporter expression in human skin in vivo and effect cell volume regulation in vitro. We hypothesize that with age, the osmoregulation of skin cells is disrupted, which could lead to impaired water homeostasis and impact skin's ability to repair when exposed to stress. Consequently, this could explain the increased incidence of dry skin (xerosis) and impaired ability to heal observed in elderly populations (Ghadially et al., 1995; White‐Chu & Reddy, 2011). In addition, it is well understood that UVR increases the susceptibility for barrier disruption via alterations to tight junction proteins, the epidermal calcium gradient, and TEWL (Jiang et al., 2007; Yuki et al., 2011). Therefore, protecting against age‐ and/or UV‐induced damage of osmolyte function could act to prevent skin dehydration and barrier disruption.

**Figure 6:**
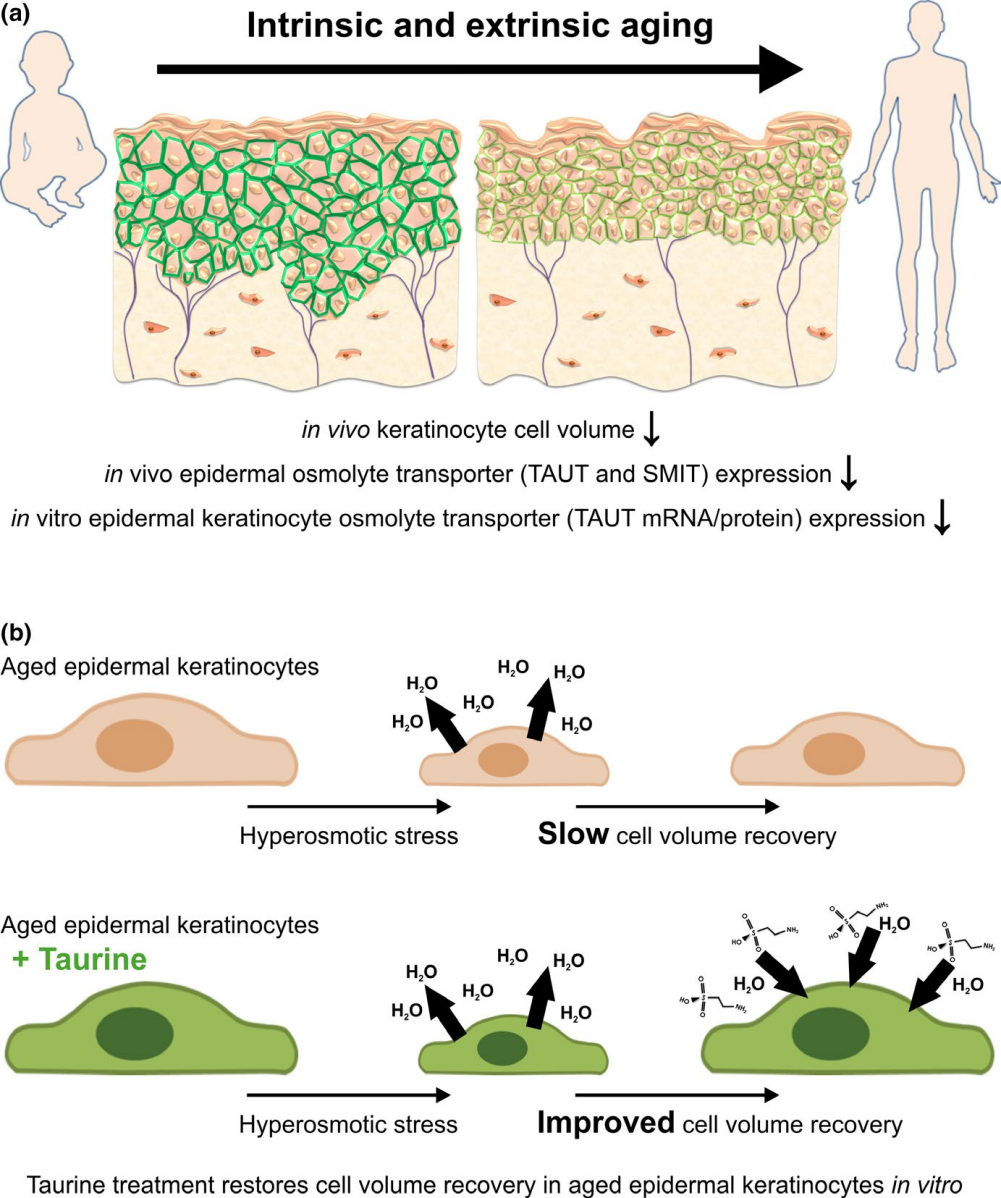
Schematic summary of osmolyte‐mediated cell volume response in aging skin. (a) Schematic summary of changes in human skin during intrinsic and extrinsic aging including reduced cell volume of epidermal keratinocytes in vivo, downregulation of osmolyte transporter proteins TAUT and SMIT in vivo, and downregulation of TAUT mRNA and protein in vitro. (b) Schematic summary of the taurine‐mediated improved cell volume regulatory mechanism in an in vitro model of aged epidermal keratinocytes. SMIT, sodium‐coupled myoinositol transporter; TAUT, taurine transporter

The reduced expression of key organic osmolyte transporters (specifically TAUT) in aged NHEKs (Figure 4), along with reduced keratinocyte cell size and loss of TAUT in aged human skin (Figure 2)—even prior to the effects of external stressors—may explain the lack of cell volume regulatory responses observed in aged NHEKs when exposed to physiological stressors in vitro (Figure 6). Crucially, addition of taurine to aged cells significantly improved rate of cell volume recovery. Consequently, we hypothesize that keratinocytes within young skin possess an enhanced capacity for cell volume regulation that allows for rapid responses to stress‐induced alterations to homeostatic conditions. Moreover, we suggest that with age this capacity is diminished leading to less efficient cell volume regulation and thus, addition of organic osmolytes, especially taurine, may be a way to preserve cell volume homeostasis (Figure 6).

The data presented here demonstrate that human epidermal keratinocytes do indeed possess an osmolyte strategy that allows cell volume regulation to take place in vivo. This study shows that cell volume and osmolyte transporter expression are negatively impacted by age and both chronic and acute UVR exposure in human skin. In conclusion, we suggest that organic osmolytes, specifically taurine, could play a key role in age‐related alterations to skin structure and function. Therefore, understanding the mechanism of action of taurine in water homeostasis control is fundamental to development of novel strategies for skin hydration management and to our understanding of the pathophysiology of skin aging.

## 4 EXPERIMENTAL PROCEDURES

### 4.1 Human skin sample collection

All human skin samples were collected from healthy volunteers recruited at the Salford Royal Teaching Hospital (Salford Royal NHS Foundation Trust; UK) with approval granted by the North West Research Ethics Committee. The study protocol for each cohort is summarized in Supplementary Materials and Methods with all donor details provided in Table S1. All samples were obtained with full written and informed consent and stored under license (Human Tissue Act [2004]; The University of Manchester, UK).

### 4.2 Immunohistochemistry, imaging, and analysis

#### 4.2.1 Immunofluorescence and histology

Skin samples were embedded in optimal cutting temperature (OCT; Miles Laboratories) compound and kept at −80°C. 7‐µm sections were prepared using a Bright OTF cryostat (Bright Instruments Ltd) and collected on Superfrost^®^ plus slides (ThermoFisher Scientific).

Immunofluorescence was carried out using the following primary antibodies rabbit anti‐SLC6A12 (dilution 1:100; catalogue #HPA034973; Sigma‐Aldrich), rabbit anti‐SLC6A6 (dilution 1:50; catalogue #HPA015028; Sigma‐Aldrich), rabbit anti‐SLC2A13 (dilution 1:1,000; catalogue #BMP026; MBL International), rabbit anti‐SLC5A3 (dilution 1:2000; catalogue #ABS518; Millipore), mouse anti‐e‐cadherin (dilution 1:500; catalogue #ab1416; Abcam), mouse anti‐K15 (dilution 1:1,000; catalogue #ab80522; Abcam), mouse anti‐CD31 (dilution 1:100; catalogue #M0823; Dako), and mouse anti‐vimentin (dilution 1:500; catalogue #M0725; Dako) as described in Supplementary Materials and Methods (Appendix S1). Finally, slides were left to dry overnight prior to imaging. Negative controls were included in every experiment by omission of the primary antibody.

### 4.3 Imaging and analysis

The Olympus BX53 upright microscope (Olympus) was used for all imaging. Negative controls were used to determine exposure settings for immunofluorescence. All images were taken under the same magnification and exposure conditions for each experiment. All images were analyzed using ImageJ software (Abràmoff, Magalhães, & Ram, 2004).

Quantitative immunohistomorphometry was conducted to measure mean fluorescence intensity per field of view on a defined anatomical areas of the skin, that is, the epidermis. The mean intensity of the epidermis was measured in three fields of view (×20 lens) per section, three skin sections per donor, in at least five donors (Figures 2, 3, and S2). For cell volume/area and diameter assessment, 10 cells were measured in a column across all cell layers of the epidermis, three columns of cells were measured per field of view. This was also carried out in three fields of view per section, three skin sections per donor, in at least five donors, giving a total of 270 cells analyzed per donor (Figure 2).

### 4.4 Single‐cell live imaging

#### 4.4.1 Normal human epidermal keratinocyte (NHEK) culture

Primary NHEKs from 3 young (7–11 years) and 3 aged (>50 years) donors were obtained from Promocell. Cells were cultured according to manufacturer's instructions using supplemented keratinocyte growth media. Once at ~80% confluence, cells were seeded in 35‐mm Ibidi dishes at a density of 5 × 10^3^ for single‐cell live imaging.

#### 4.4.2 Single live‐cell imaging

For the hyperosmotic stress cell experiments (Figure 4 and S4) on the day of live imaging, NHEKs were treated with 5 µM calcein (in PBS) for 15 min at 37°C then washed with PBS for 30 min at 37°C. Cells were loaded onto the microscope and exposed to hyperosmotic stress in the absence and presence of organic osmolytes. For isosmotic conditions, normal NHEK growth medium was used with an osmolarity of ~300 mOsm. For hyperosmotic conditions, mannitol was added to normal NHEK growth medium to gain an osmolarity of ~500 mOsm. A 500 mM stock solution of each osmolyte was prepared then added to media to obtain a 5 mM final concentration of betaine, myoinostiol, or taurine. Cells were imaged using the Leica SP8 gSTED 3× super resolution microscope and LasX software. Imaging for each cell was carried out in a 32‐min cycle of 5 min in isosmotic conditions, 15 min hyperosmotic conditions, and 12 min in isosmotic conditions. An image was captured in the x‐z direction every 10 s and media was pumped in and out of the petri dish at a constant rate of 1 ml per minute throughout the video. For each donor sample (three young and three aged), 10 cells were imaged for 32 min per condition.

For the SSR cell experiments, cells were exposed to 80mJ SSR and then incubated in the absence and presence of organic osmolytes for 1 and 4 hr. Cells were then imaged at each timepoint on the Leica SP8 gSTED 3× super resolution microscope. Again, 10 cells were imaged at 1 and 4 hr per donor sample (3 young and 3 aged).

#### 4.4.3 Cell volume analysis

Live‐cell imaging videos were opened in ImageJ for analysis. Cell volume was measured for each image captured via particle analysis. These data were then used to calculate rate of regulatory volume increase (RVI), maximum percentage cell volume decrease, and percentage cell volume regain.

### 4.5 Quantitative real‐time PCR (qRT–PCR)

RNA was extracted from NHEK cell pellets from each donor using the RNAeasy mini kit (Qiagen) according to manufacturer's protocol. RNA was converted to cDNA using the Tetro cDNA synthesis kit (Bioline) according to manufacturer's protocol. For each sample, qRT–PCR was performed in triplicate using Taqman probes for the osmolyte transporters *BGT‐1, HMIT, SMIT*, or *TAUT* (see Appendix S1), and reactions were performed on the StepOnePlus RT–PCR machine. Relative percentage mRNA expression was calculated for each gene of interest against the housekeeping gene (*PPIA*) using the ΔCT method.

### 4.6 Western blot analysis

Total protein was isolated from NHEKs from each donor as described in Supplementary Materials and Methods (Appendix S1).

Protein samples for Western blotting were prepared by adding 15 µg of protein with LDS sample buffer (4×; catalogue #NP0007, Invitrogen), reducing agent (10×; catalogue #NP0009, Invitrogen), and extraction buffer to make up a total loading volume of 12 µl per well. Samples were vortexed and heated to 85°C for 10 min. Protein samples were then run on a NuPAGE™ 4%–12% Bis‐Tris Protein Gel (Invitrogen) at 185 V for 50 min. Next, semiwet transfer to nitrocellulose membranes was carried out using the Bio‐Rad Trans‐Blot Turbo Transfer system (Bio‐Rad). Next, blocking was carried out using 5% milk solution in 1X TBS + 0.5% Tween‐20 for 1 hr. Membranes were incubated overnight at 4°C with the following primary antibodies: rabbit anti‐SLC6A12 (dilution 1:1,000; catalogue #HPA034973; Sigma‐Aldrich), rabbit anti‐SLC6A6 (dilution 1:1,000; catalogue #HPA015028; Sigma‐Aldrich), rabbit anti‐SLC2A13 (dilution 1:1,000; catalogue #BMP026; MBL International), rabbit anti‐SLC5A3 (dilution 1:1,000; catalogue #ABS518; Millipore), and rabbit anti‐β actin (dilution 1:20,000; catalogue #ab8227; Abcam). On day 2, membranes were washed in 1X TBS + 0.5% Tween‐20 before incubation with goat anti‐rabbit HRP secondary antibody (dilution 1:5,000; catalogue #170‐6515; Bio‐Rad). For detection, membranes were incubated with Amersham ECL Western blotting detection reagent (1:1 ratio; GE Healthcare Life Sciences) for 2 min before exposing film to the membrane in a dark room and developing using the JP 33 Film Processor. Densitometry analysis was performed using ImageJ.

### 4.7 Statistical analysis

All statistical analysis was carried out using GraphPad Prism 7.0. First, data were assessed for variance and normal distribution to decide whether a parametric or nonparametric test should be used. Then, Student's *t* test was used for analysis between two groups (Figure 4 and S3), one‐way analysis of variance (ANOVA) was used for analysis between three or more groups (Figures 3, 4, 5, and S4), or two‐way ANOVA was used for analysis of two variables (Figure 2 and S2). Statistical significance was determined by differences with a *p*‐value <.05.

## CONFLICT OF INTEREST

The authors have no conflict of interest to declare.

## AUTHOR CONTRIBUTIONS

ARF performed the experiments and analyzed the data. CEC provided experimental advice and helped with data analysis. CAO and REBW conceived and supervised the study, providing experimental and analysis advice. All authors were involved in writing and editing the manuscript with final approval of the submitted versions.
