# Supplementary material for: Osmolyte transporter expression is reduced in photoaged human skin: Implications for skin hydration in aging
Source: Aging Cell. 2019 Nov 26;19(1):e13058. doi: 10.1111/acel.13058 (PMC6974728; doi:10.1111/acel.13058)
Supplement: Supplementary file 1 [file ACEL-19-e13058-s001.pdf]

Supporting information

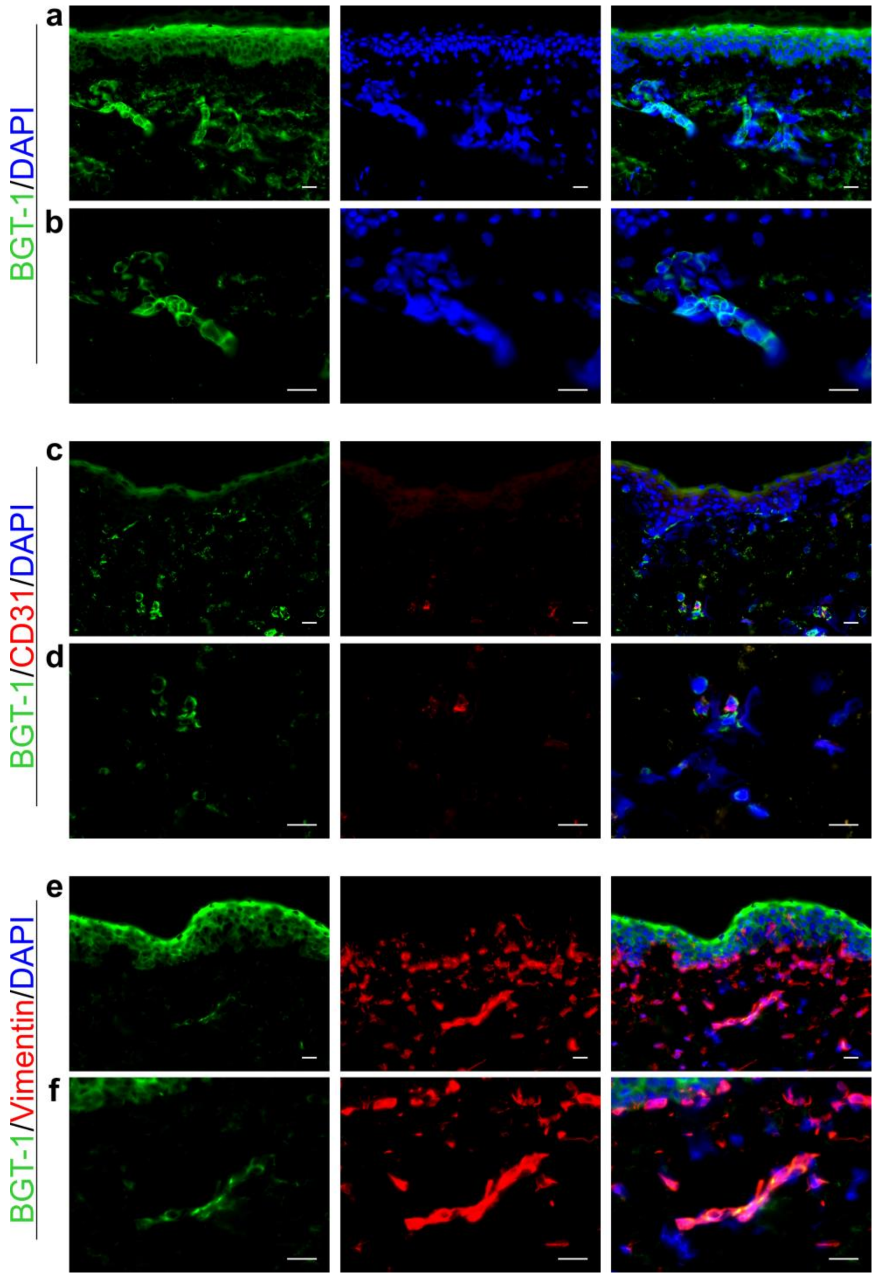

Figure S1. BGT-1 is co-expressed with CD31 and vimentin in dermal cells of human skin.

(**a-b**) BGT-1 protein is expressed as a gradient throughout the epidermis and in the dermis of human skin. BGT-1 is co-expressed by dermal cells that also express (**c-d)** CD31 and (**e-f**) vimentin. Scale bars = 20µm. BGT-1, betaine transporter; CD31, cluster of differentiation 31; DAPI, 4′,6-diamidino-2-phenylindole.

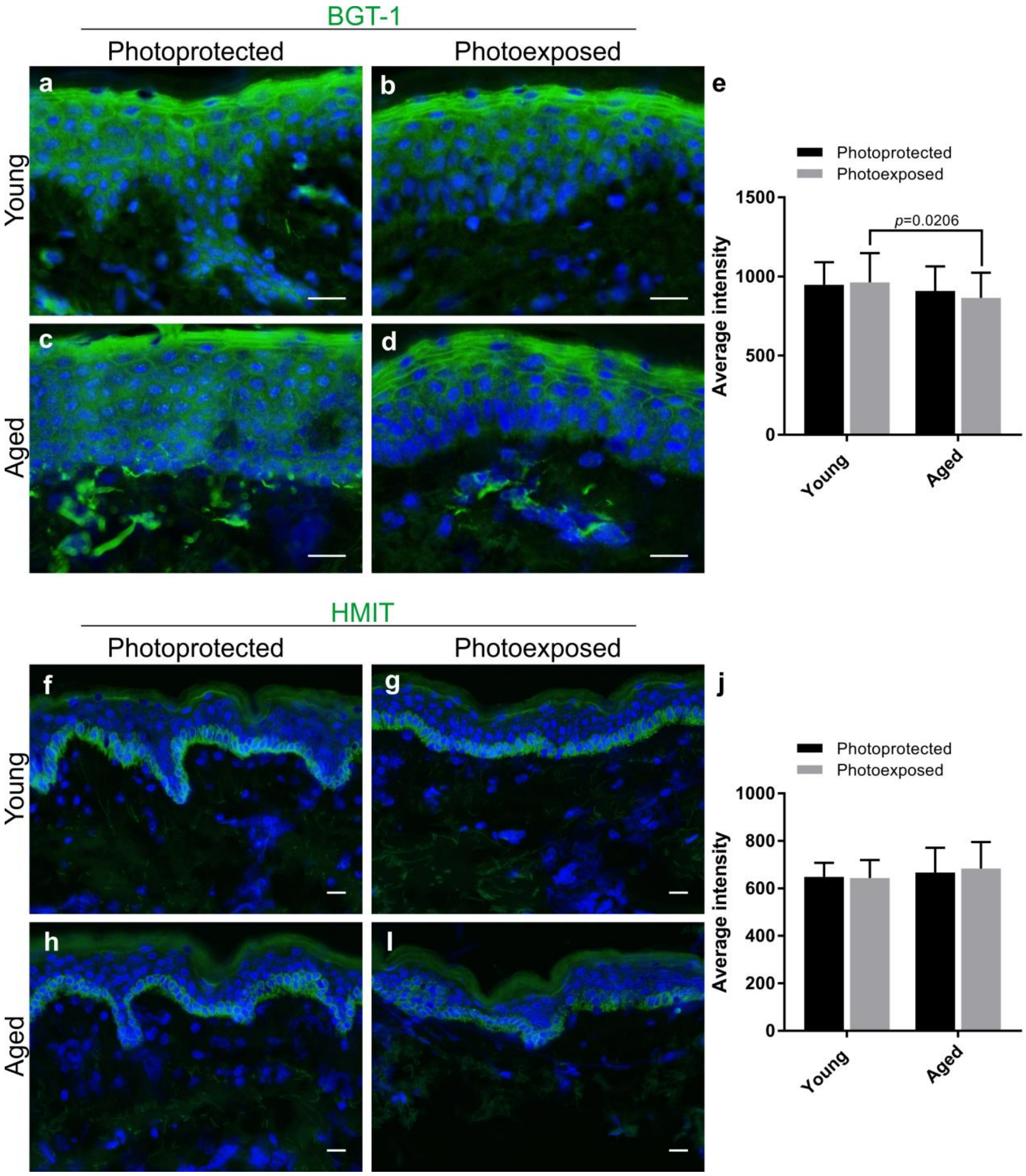

Figure S2. BGT-1 and HMIT protein expression in young and aged, photoprotected and photoexposed human skin.

Immunofluorescence for (**a-d**) BGT-1 and (**f-i**) HMIT in and aged photoprotected and photoexposed skin. Immunofluorescence intensity analysis showed (e) BGT-1 protein expression is downregulated in aged photoexposed skin compared to young photoexposed skin (p=0.0206). (j) HMIT protein expression did not change with age and photoexposure. Data expressed as mean ± SD (two-way ANOVA), young n=5, aged n=6. Scale bars = 20μm. BGT-1, betaine transporter; HMIT, hydrogen-coupled myoinositol transporter.

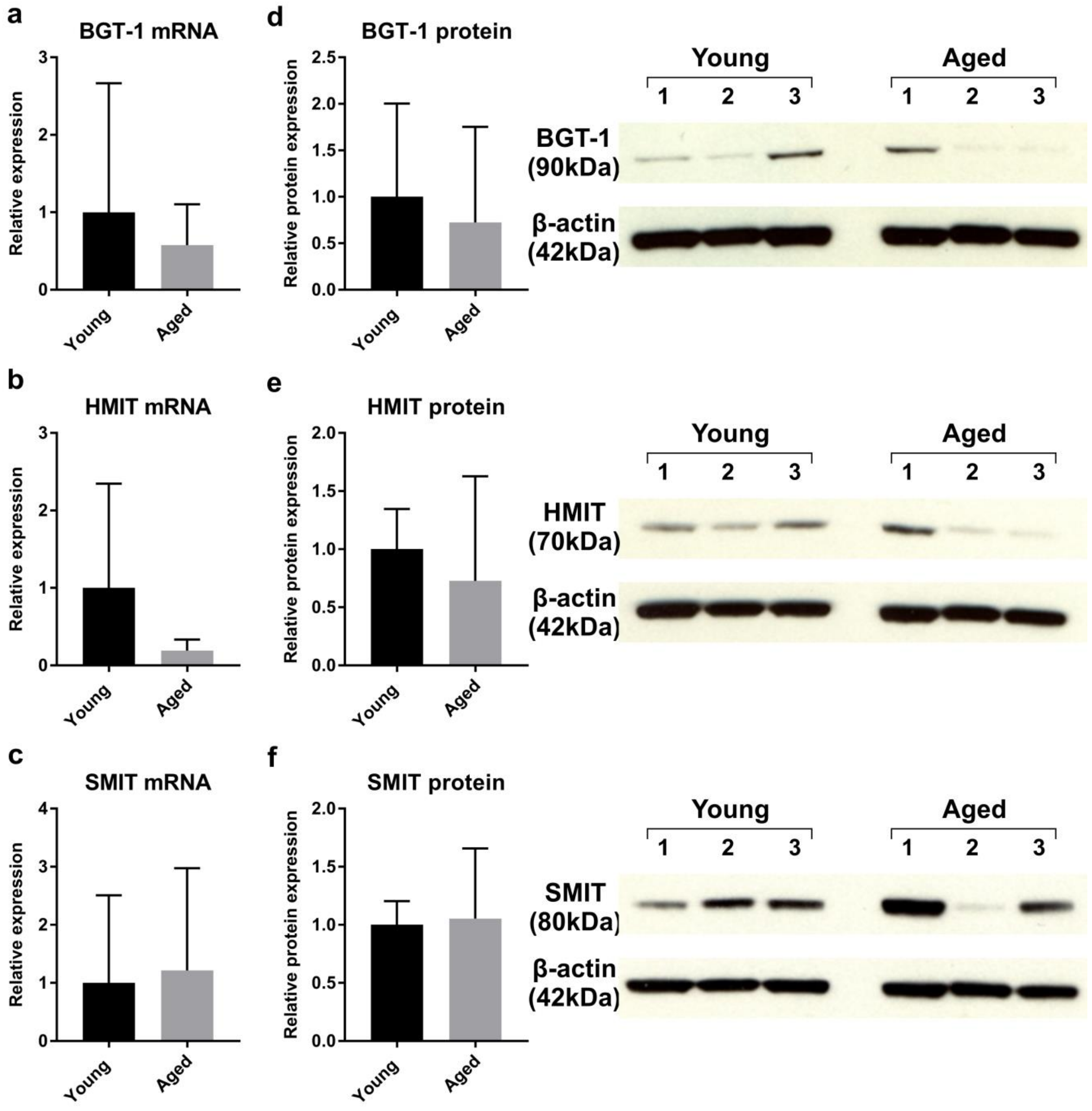

Figure S3. BGT-1, HMIT and SMIT mRNA and protein expression in young and aged NHEKs.

(**a-c**) BGT-1, HMIT and SMIT mRNA expression quantified via qPCR. (**d-f**) BGT-1, HMIT and SMIT protein expression quantified via Western blot. Data expressed as mean ± SD (student t-test), n= 3 young and 3 aged donors. BGT-1, betaine transporter; HMIT, hydrogen-coupled myoinositol transporter; NHEKs, normal human epidermal keratinocytes; SMIT, sodium-coupled myoinositol transporter.

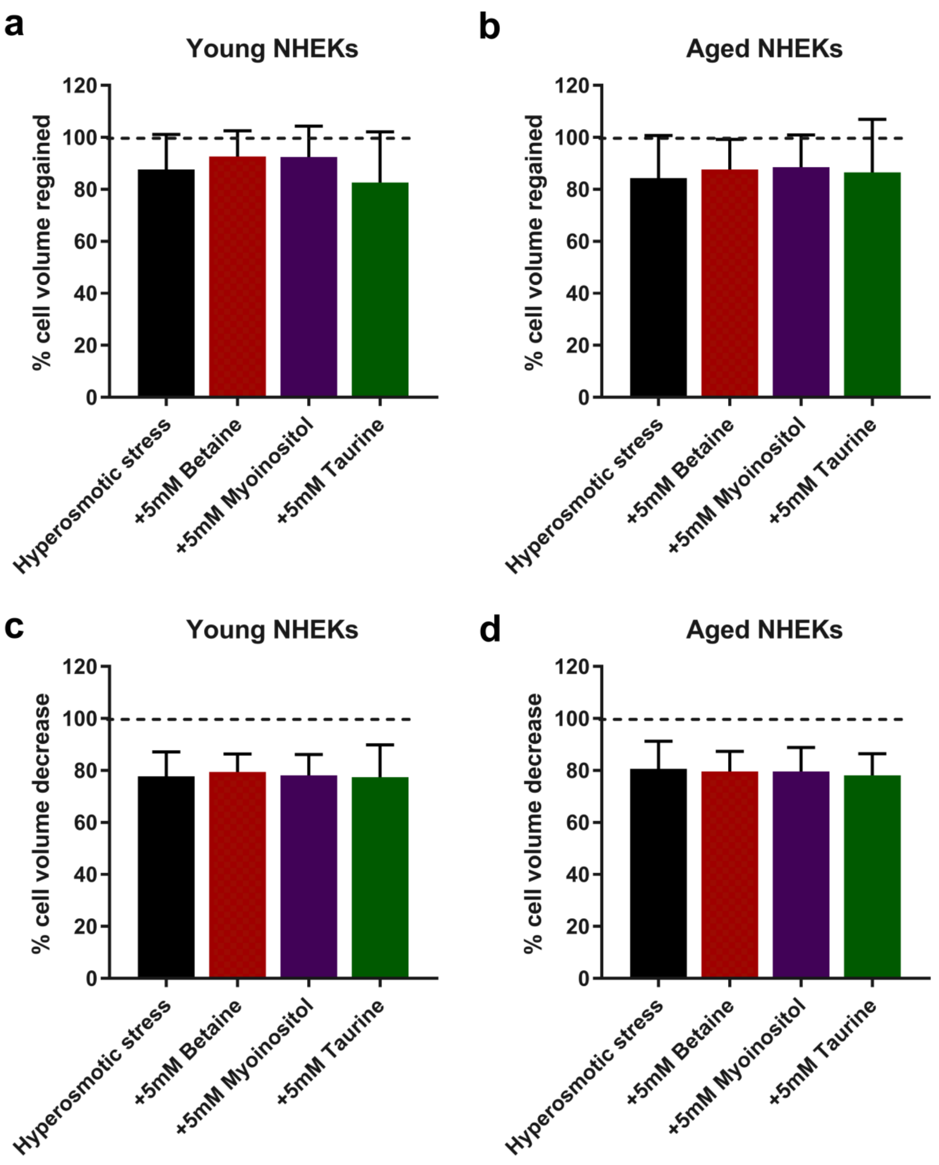

Figure S4. Percentage cell volume regain and cell volume decrease in young and aged NHEKs.

Young and aged NHEKs exposed to hyperosmotic conditions in the absence or presence of organic osmolytes were captured using single cell live imaging. Cell images were assessed for (**a-b**) percentage cell volume regained and (**c-d**) maximum cell volume decrease. Data expressed as mean ± SD (one-way ANOVA), n=10 cells per donor from 3 young and 3 aged donors. NHEKs, normal human epidermal keratinocytes.

a

Study 1 – Young and aged photoprotected and photoexposed skin	
**Young donors**	
1	29 Female
2	30 Male
3	28 Female
4	28 Female
5	24 Female
**Aged donors**	
1	68 Male
2	68 Female
3	70 Female
4	67 Male
5	67 Male
6	77 Female

b

Study 2 – SSR irradiation study (80mJ)	
1	34 Male
2	44 Male
3	38 Male
4	30 Male
5	36 Female

Table S1. Donor details for human skin studies.

Human skin samples were collected from healthy volunteers recruited into two different cohorts. (a) Cohort one included 5 young (mean age 27.8 years) and six aged (mean age 69.5 years) donors. (b) Cohort two included 5 individuals with a mean age 36.4 years.

Supplementary Materials and Methods

Study cohorts and protocols

Cohort one (ref#15464) included five young (18-30 years; 3 female and 2 male) and six aged (>65 years; 3 female and 3 male) volunteers. Each volunteer had two skin biopsies taken, one from photoprotected buttock and one from photoexposed forearm. For cohort two (ref#15439), five volunteers (average age 37.25 years; 1 female and 4 male) were exposed to acute solar simulated radiation (SSR) at eight times standard erythema dose (SED; 80 mJ) at the photoprotected buttock site. A skin biopsy was taken from an unirradiated site (baseline) then at 1-, 3- and 72-hours post-SSR exposure.

Immunofluorescence

For all immunofluorescence carried out for the organic osmolyte transporters (single or dual stains) sections were air dried for 10 minutes at room temperature (RT) and fixed in chilled 100% acetone for 15 minutes. For single immunofluorescence of e-cadherin, sections were fixed in chilled 4% PFA for 20 minutes. Blocking was carried out using 2.5% normal horse serum for 30 minutes at RT. Tris-buffered saline solution was used for all wash steps. Primary antibodies were incubated overnight at 4°C, if carrying out dual immunofluorescence primary antibodies were incubated together simultaneously. On day 2 for all single immunofluorescence, VectaFluor™ excel amplified DyLight® 488 anti-rabbit IgG or 594 anti-mouse IgG kit was used (catalogue #DK-1488; Vector Laboratories) applying the amplifier for 15 minutes at RT, with subsequent application of VectaFluor DyLight-488 secondary antibody (30 minutes; RT). On day 2 for dual immunofluorescence, VectaFluor™ duet immunofluorescence double labeling kit, DyLight® 488 anti-rabbit

(green)/DyLight 594 anti-mouse (red) was used (catalogue #DK8818; Vector Laboratoies) applying the secondary antibodies for 30 minutes at RT. 4′,6-diamidino-2-phenylindole (DAPI; ThermoFisher Scientific) was used to counterstain nuclei and Fluoromount G (Southern Biotech; Birmingham, AL, USA) was used to mount slides with coverslips.

qRT-PCR

The Taqman probes used to carry out qRT-PCR were BGT-1 (Hs_00758246_m1), TAUT (Hs_00161778_m1), HMIT (Hs_00369423_m1) or SMIT (Hs_00272857_s1) for the genes of interest and PPIA (Hs_04194521_s1) for the housekeeping gene.

Protein extraction

NHEKs were grown in a 12-well plate until confluency for protein extraction. Total protein was isolated from NHEKs using extraction buffer of the following composition in 100ml of distilled water: 120mM sodium chloride, 25mM HEPES, 1% triton X-100, 25mM sodium fluoride, 1mM sodium orthovanadate, 0.2% SDS and 0.1mg/ml protease inhibitor cocktail (catalogue #P8340; Sigma). Cells were washed twice with ice cold PBS then 100µl of extraction buffer was added to each well and cell scraping was used to collect the cells in the buffer. The lysate was then kept on ice for 30 minutes before sonication at half maximum speed for 5 seconds. Samples were then centrifuged at 13000rpm for 20 minutes and the supernatant was collected. Protein concentration was quantified using the Invitrogen Qubit 4 Fluorometer (ThermoFisher Scientific).
